# Phylowood: interactive web-based animations of biogeographic and phylogeographic histories

**DOI:** 10.1093/bioinformatics/btt635

**Published:** 2013-11-04

**Authors:** Michael J. Landis, Trevor Bedford

**Affiliations:** ^1^Department of Integrative Biology, UC Berkeley, Berkeley, CA 94720, USA, ^2^Institute of Evolution, School of Biological Sciences, University of Edinburgh, Edinburgh EH9 3JT, UK and ^3^Vaccine and Infectious Disease Division, Fred Hutchinson Cancer Research Center, Seattle, WA 98109, USA

## Abstract

**Summary:** Phylowood is a web service that uses JavaScript to generate in-browser animations of biogeographic and phylogeographic histories from annotated phylogenetic input. The animations are interactive, allowing the user to adjust spatial and temporal resolution, and highlight phylogenetic lineages of interest.

**Availability and implementation:** All documentation and source code for Phylowood is freely available at https://github.com/mlandis/phylowood, and a live web application is available at https://mlandis.github.io/phylowood.

**Contact:** mlandis@berkeley.edu

## 1 INTRODUCTION

The fields of phylogeography and biogeography study the processes that give rise to the observed geographical distributions of life. Methods to infer migration processes and reconstruct ancestral geographical distributions in a phylogenetic context have recently enjoyed increased popularity (Landis *et al.*, 2013; Lemey *et al.*, 2009; Ree and Smith, 2008). The resulting ancestral reconstructions are inherently high dimensional; they describe distributions across space, time and phylogenetic lineage and consequently can be difficult to interpret. Here, we introduce Phylowood, a web utility that generates interactive animations to facilitate the exploration and summarization of such complex reconstructions.

Phylowood takes phylogeographic output from BEAST (Drummond *et al.*, 2012) or biogeographic output from BayArea (Landis *et al.*, 2013) in the form of NEWICK trees with internal nodes annotated with inferred ancestral locations. Phylowood plots the reconstructed geographic distributions and explores temporal dynamics through animation. Although similar in basic approach to the program SPREAD (Bielejec *et al.*, 2011), Phylowood is designed for ease-of-use and frictionless sharing. It is entirely implemented within the web browser, requiring no further software installation.

## 2 USE AND IMPLEMENTATION

**Fig. 1.**
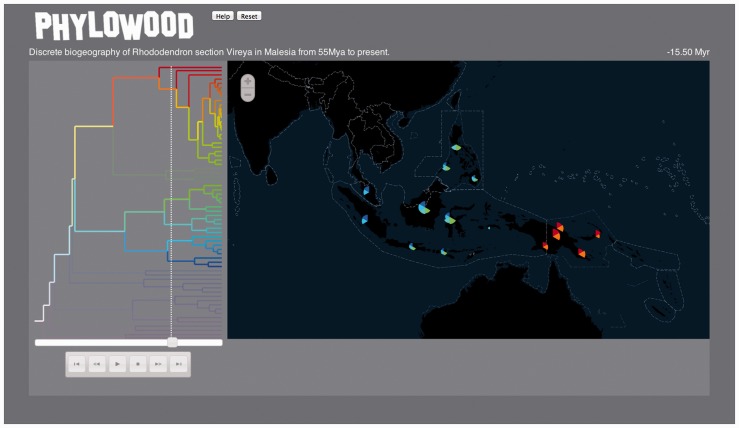
Sample still frame from Phylowood. The results shown are from the discrete biogeographic analysis of *Rhododendron* section *Vireya* throughout Malesia using BayArea. Much of the phylogenetic tree has been masked using mouse-issued commands, leaving two clades and their shared ancestry unmasked. The media slider indicates the current position of the animation with respect to the time-calibrated phylogeny for which time six unmasked lineages are animated. For the current animation time, each extant lineage is allocated an equal width slice of the pie. For each color, the depth of the slice indicates the approximate marginal posterior probability of the lineage occupying the area at that time. Pie slices are sorted phylogenetically, making the relative position of absent slices informative. Consulting the geography panel, we find the taxa from the top clade appear to be allopatric with respect to taxa from the bottom clade at ∼15.5 Mya. The interactive animation is available at http://mlandis.github.io/phylowood/?url=examples/vireya.nhx

Phylowood has two primary display panels: the phylogeny panel and the geography panel (Fig. 1). The phylogeny panel (left) contains a time-calibrated phylogeny, where lineages are assigned unique colors that reflect phylogenetic proximity. The geography panel (right) contains colored area markers corresponding to phylogenetic lineages that specify discrete or continuous geographic distributions. Below the tree, standard media buttons control the animation speed, direction and location. The animation time slider shows the current time point and indicates the lineages that exist at this time, which comprise targets for animation.

Filtering out uninteresting data is a key to exploration and summarization. Phylowood allows users to mask, unmask and highlight sets of branches using simple doubleclick, click and mouseover events through either the phylogeny or geography displays. For example, mask and unmask commands may be used to remove all but 10 lineages from a dataset containing 1000 taxa. Mouseover events provide information about highlighted lineage and help users match phylogenetic lineages to their geographic counterparts.

The geography panel contains a dynamic map, capable of zooming and panning, and area markers representing geographic distributions at the current time point for unmasked phylogenetic lineages. To reflect the underlying model assumptions that produced the ancestral area reconstructions, we allow several styles: continuous phylogeography, discrete phylogeography and discrete biogeography. For continuous phylogeographic animations, the reconstructed state for each node in the phylogeny is a unique latitude and longitude. For discrete phylogeography and discrete biogeography, each node’s reconstructed state corresponds to geographical coordinates representative of a number of the specified discrete areas. For a given phylogenetic branch, the radius of a corresponding geographic marker is proportional to the value assigned to that geographic state. Depending on reconstruction methodology, such values may represent a posterior probability, confidence metric or parsimony score. Intermediate values along phylogenetic branches are interpolated between reconstructed internal phylogenetic nodes. In the discrete phylogeographic or biogeographic scenarios, interpolation represents the e.g. posterior probability of assignment along a branch of the phylogeny. In the continuous phylogeographic scenario, interpolation represents the reconstructed continuous location along a branch of the phylogeny. Similar to the branches in the phylogenetic panel, area markers respond to the highlight mouseover command.

We provide several demonstration datasets, encompassing continuous phylogeographic, discrete phylogeographic and discrete biogeographic scenarios. Users can easily animate their own datasets through the web service. Additionally, the hosting of code and data on GitHub allows users to easily fork the repository and provide one-click links to their own custom visualizations. This is intended to make sharing of specific visualizations easy. Phylowood was developed in JavaScript and thus compatible with any HTML5-compliant web browsers with no installation required. Scalable Vector Graphics objects are managed and animated using D3.js (Bostock *et al.*, 2011). Map tiles are fetched from Cloudmade using Polymaps. Source code is published under the Massachusetts Institute of Technology Software License and made freely available at http://github.com/mlandis/phylowood.

Animations are generated from a NEXUS format file, specifying the animation settings, geographical coordinates and a New Hampshire extended format (NEWICK) tree annotated with ancestral area values. We provide Ruby scripts to convert BEAST output to Phylowood format, with more to be developed on demand. BayArea natively produces Phylowood format files.

## 3 EXAMPLE: *VIREYA*

*Rhododendron* section *Vireya* is a group of flowering plants distributed throughout Malesia (Fig. 1). From the posterior probabilities of ancestral range reconstructions, Landis *et al.* (2013) infer the ancestor common to Malesian *Vireya* originated on continental Asia, concurring with results reported in Webb and Ree (2012). Approximately 35–45 Mya, a single *Vireya* lineage first colonized islands east of both Wallace’s and Lydekker’s lines, known barriers to dispersal events. Interestingly, descendants of the lineage remain strictly to the east of Lydekker’s line, whereas all remaining taxa remain to its west. This scenario is made evident using Phylowood by filtering and animating these otherwise high-dimensional data: (i) masking clades whose colors appear west of Lydekker’s line, just west of New Guinea, (ii) panning the map over Malesia, (iii) filtering out areas with low probability and (iv) beginning the animation at 45 Mya.

## 4 CONCLUSION

Phylogeographic and biogeographic methods combine genetic and geographic data across individuals or across species to infer complex spatiotemporal processes. Joint phylogenetic and geographic reconstructions produced by these methods benefit from sophisticated visualization techniques that highlight connections between geography and genotype. As phylogeographic and biogeographic datasets continue to increase in size and complexity, harnessing appropriate visualization techniques will become increasingly important. We present Phylowood as a method to explore and share geographically tagged phylogenies.

To learn more about Phylowood’s features, visit http://github.com/mlandis/phylowood/wiki for help and tutorials.
